# Effluent solids recirculation to municipal sludge digesters enhances long-chain fatty acids degradation capacity

**DOI:** 10.1186/s13068-021-01913-1

**Published:** 2021-03-04

**Authors:** Sepehr Shakeri Yekta, Tong Liu, Thuane Mendes Anacleto, Mette Axelsson Bjerg, Luka Šafarič, Xavier Goux, Anna Karlsson, Annika Björn, Anna Schnürer

**Affiliations:** 1 Department of Thematic Studies-Environmental Change, Linköping University, 58183 Linköping, Sweden; 2 Biogas Research Center, Linköping University, 58183 Linköping, Sweden; 3 Department of Molecular Sciences, Swedish University of Agricultural Sciences, Uppsala BioCenter, 75007 Uppsala, Sweden; 4 Post Graduate Program in Plant Biotechnology and Bioprocesses, Federal University of Rio de Janeiro, Rio de Janeiro, 21941-901 Brazil; 5 Environmental Research and Innovation Department, Luxembourg Institute of Science and Technology, 4422 Belvaux, Luxembourg; 6 Scandinavian Biogas Fuels AB, 11160 Stockholm, Sweden

**Keywords:** Anaerobic digestion, Primary and activated sewage sludge, Microbial community, Oleate, Feeding frequency, Sulfide

## Abstract

**Background:**

Slow degradation kinetics of long-chain fatty acids (LCFA) and their accumulation in anaerobic digesters disrupt methanogenic activity and biogas production at high loads of waste lipids. In this study, we evaluated the effect of effluent solids recirculation on microbial LCFA (oleate) degradation capacity in continuous stirred-tank sludge digesters, with the overall aim of providing operating conditions for efficient co-digestion of waste lipids. Furthermore, the impacts of LCFA feeding frequency and sulfide on process performance and microbial community dynamics were investigated, as parameters that were previously shown to be influential on LCFA conversion to biogas.

**Results:**

Effluent solids recirculation to municipal sludge digesters enabled biogas production of up to 78% of the theoretical potential from 1.0 g oleate l^−1^ day^−1^. In digesters without effluent recirculation, comparable conversion efficiency could only be reached at oleate loading rates up to 0.5 g l^−1^ day^−1^. Pulse feeding of oleate (supplementation of 2.0 g oleate l^−1^ every second day instead of 1.0 g oleate l^−1^ every day) did not have a substantial impact on the degree of oleate conversion to biogas in the digesters that operated with effluent recirculation, while it marginally enhanced oleate conversion to biogas in the digesters without effluent recirculation. Next-generation sequencing of 16S rRNA gene amplicons of bacteria and archaea revealed that pulse feeding resulted in prevalence of fatty acid-degrading *Smithella* when effluent recirculation was applied, whereas *Candidatus Cloacimonas* prevailed after pulse feeding of oleate in the digesters without effluent recirculation. Combined oleate pulse feeding and elevated sulfide level contributed to increased relative abundance of LCFA-degrading *Syntrophomonas* and enhanced conversion efficiency of oleate, but only in the digesters without effluent recirculation.

**Conclusions:**

Effluent solids recirculation improves microbial LCFA degradation capacity, providing possibilities for co-digestion of larger amounts of waste lipids with municipal sludge.

**Supplementary Information:**

The online version contains supplementary material available at 10.1186/s13068-021-01913-1.

## Background

Increasing biogas production is of strategic importance for achieving Sweden’s goal of zero net greenhouse gas emissions by 2045 [1]. The national biogas strategy has set the goal of annual biogas energy use of 15 TWh by 2030, which requires a substantial increase in Sweden’s biogas production capacity [2]. Anaerobic digester units at wastewater treatment plants (WWTP) account for approximately half of the existing biogas production facilities in Sweden and their existing capacity can be used for more biogas production through co-digestion of energy-rich organic wastes [3, 4]. In this context, waste lipids are regarded as attractive co-substrates because of their high methane potential and energy density [5]. However, formation of long-chain fatty acids (LCFA) during anaerobic degradation of waste lipids and potential inhibitory effects on methanogenic activity make the use of waste lipids uncertain and challenging [6].

In anaerobic digesters, lipids are hydrolyzed by microbial lipases to glycerol and LCFA (e.g., palmitoleic, palmitic, stearic, oleic, and linoleic acid) [7]. Further microbial degradation of LCFA is carried out via the cyclic β-oxidation pathway. In each cycle, the LCFA chain is shortened by two carbon atoms, acetate is produced from acetyl-CoA, and hydrogen is released coupled to FAD/FADH_2_ and NAD^+^/NADH redox reactions [8]. Hydrogen production via FADH_2_ and NADH oxidation are endergonic, necessitating establishment of low hydrogen partial pressure for LCFA β-oxidation [8]. The low hydrogen partial pressure is established by syntrophic association of LCFA-degrading bacteria with hydrogen- and/or formate-utilizing microorganisms in anaerobic digesters, where co-occurrences and activity of β-oxidizers (e.g., families Syntrophomonadaceae and Syntrophaceae) together with hydrogenotrophic methanogens (e.g., *Methanoculleus*, *Methanobacterium*, and *Methanothermobacter*) have been identified as the main contributors to LCFA degradation [9].

Different threshold concentrations for partial and complete inhibition of methane formation by LCFA have been reported for different methanogenic systems. Concentration of LCFA (caprylic, capric, lauric, myristic, and oleic acids) in the range of ~ 0.9 and 1.4 g l^−1^ in batch assays that were inoculated with granular sludge resulted in a 50% reduction of the activity of acetolactic methanogens, compared to the similar system without LCFA [10]. Oleic and stearic acid concentrations of 0.5 and 1.0 g l^−1^, respectively, led to a complete inhibition of microbial growth during thermophilic digestion of manure in anaerobic batch assays [11]. Similarly, inhibition of the methane formation from microbial communities originated from sludge digesters at the presence of 1.0 g l^−1^ LCFA (e.g., oleic and stearic acids) have been observed under both mesophilic and thermophilic conditions [12, 13].

Slow kinetics of LCFA degradation compared with lipid hydrolysis often leads to LCFA accumulation in digesters with high loads of waste lipids, where the LCFA accumulation in turn may lead to extensive LCFA adsorption on solid particles, sludge floatation, foaming, microbial inhibition, and ultimately process failure [5, 6]. In this respect, retention time of lipid-rich substrates in digesters has a profound effect on degradation efficiency of LCFA and process stability [14, 15]. Prolonged retention time of LCFA-amended sludge (e.g., by applying a batch-mode operation after a period of continuous feeding of lipids), where the accumulated LCFA are mainly confined to the solid fraction, results in higher conversion efficiency of LCFA [6, 16, 17]. LCFA feeding frequency has also been identified as an important parameter for kinetics and microbial stability during anaerobic degradation of LCFA, where pulse feeding (instead of continuous feeding) may trigger the activity of β-oxidizing bacteria and improve LCFA degradation [18]. In a previous study of the sulfide-induced changes in methanogenic activity and turnover of anaerobic digestion intermediates, including LCFA, we observed that the microbial community in municipal sludge digesters that were exposed to sulfide was able to convert oleate to methane with a faster rate compared to those without sulfide exposure [19]. We further observed that sulfide is a selective driver for the establishment and growth of LCFA-degrading bacteria in municipal sludge digesters [16, 19].

Based on the above, this study evaluated the impact of effluent solids recirculation, as an operational approach for prolonging sludge retention, on LCFA conversion to biogas in municipal sludge digesters. The aim was to provide operating conditions for efficient co-digestion of waste lipids in municipal sludge digesters by improving the microbial LCFA degradation capacity. A second aim was to assess the influence of LCFA feeding frequency and sulfide level as important operating parameters for microbial LCFA degradation. Effluent recirculation has been shown to contribute to greater process stability in different anaerobic digestion systems, such as two-stage and dry digesters [20, 21]. To our knowledge, information on the impact of recirculating effluent solids to municipal digesters on process performance, and in particular on LCFA degradation, is not well known.

## Methods

### Experimental set-up

Six laboratory stirred-tank anaerobic reactors (Belach Bioteknik, Skogås, Stockholm, Sweden) were inoculated with sludge from a full-scale anaerobic digester at Henriksdal WWTP in Stockholm, Sweden. Primary and activated sewage sludge (PASS) from the same WWTP were collected on one occasion and stored in 10-L containers at − 20 °C. Mixtures of 80% primary sludge and 20% activated sludge (similar to the full-scale digester at Henriksdal WWTP), on a volume basis, with total solids (TS) content of 2.7 ± 0.3% of total weight and volatile solids (VS) content of 75 ± 8% of TS, were used as substrates throughout the experiment. The laboratory digesters, designated R1–R6, were operated at mesophilic conditions (37 °C), with a working volume of 6 L and an average PASS loading rate of 1.3 ± 0.1 g VS l^−1^ day^−1^, simulating the operating conditions of the full-scale digester at Henriksdal WWTP. A hydraulic retention time of 20 days was maintained in the digesters by daily feeding of 0.3 L of PASS (influent) and withdrawal of 0.3 L of digester sludge (effluent). For R4, R5, and R6, 0.15 L of the effluent was centrifuged (7000 × *g*, 10 min) and the supernatant was decanted after centrifugation. Thereafter, the remaining solid fraction (effluent solids) was recirculated, while the total influent and effluent volume of 0.3 L was maintained by compensating for volume of the recirculated solids. As a result, recirculation rates of 0.5–0.6 g TS l^−1^ day^−1^ were obtained during the start-up phase of operation (Table 1).

**Table 1:** Operating conditions of the laboratory stirred-tank anaerobic digesters (R1–R6)

	Days^a^	R1	R2	R3	R4	R5	R6
PASS loading rate (gVS l^−1^ day^−1^)	0–200	1.3 ± 0.1	1.3 ± 0.1	1.3 ± 0.1	1.3 ± 0.1	1.3 ± 0.1	1.3 ± 0.1
	201–255	0.0	0.0	0.0	0.0	0.0	0.0
Oleate loading rate (g l^−1^ day^−1^)	0–84	0.0	0.0	0.0	0.0	0.0	0.0
	85–91	0.0	0.08	0.08	0.0	0.08	0.08
	92–112	0.0	0.13	0.13	0.0	0.13	0.13
	113–130	0.0	0.25	0.25	0.0	0.25	0.25
	131–151	0.0	0.5	0.5	0.0	0.5	0.5
	152–171	0.0	1.0	1.0	0.0	1.0	1.0
Pulse feeding^b^ →	172–200	0.0	1.0	1.0	0.0	1.0	1.0
	201–255	0.0	0.0	0.0	0.0	0.0	0.0
Effluent solids recirculation rate (gTS l^−1^ day^−1^)	0–84	0.0	0.0	0.0	0.6 ± 0.0	0.5 ± 0.1	0.6 ± 0.1
	85–171	0.0	0.0	0.0	0.8 ± 0.2	0.8 ± 0.1	0.9 ± 0.2
	172–200	0.0	0.0	0.0	1.1 ± 0.1	1.2 ± 0.1	1.3 ± 0.1
	201–255	0.0	0.0	0.0	0.0	0.0	0.0
Sulfide addition (mmol l^−1^ day^−1^)	0–200	0.0	0.0	20	0.0	0.0	20
	201–255	0.0	0.0	0.0	0.0	0.0	0.0

^a^Day 0–84: start-up phase; day 85: start of oleate addition to R2, R3, R5, and R6; day 172: start of oleate pulse feeding; day 201: end of PASS and oleate feeding

^b^Addition of 2.0 g l^−1^ of oleate every second day is referred to as pulse feeding

In addition, 20 mmol S l^−1^ substrate (Na_2_S.9H_2_O in de-aerated ultrapure water) were added to digesters R3 and R6. Sulfide was added in a level to obtain an S:Fe molar ratio of 0.9 in R3 and R6, in order to ensure an excess of Fe over sulfide (data not shown), since maintaining S:Fe molar ratio < 1 allows mitigating potential process disturbances associated with sulfide inhibition in PASS digesters [19]. From day 85, oleate (C_18:1_) was fed daily (i.e., semi-continuous feeding), to R2, R3, R5, and R6, with a stepwise increase in loading rate from 0.08 to 1.0 g l^−1^ day^−1^ (Table 1) in order to avoid shock load of oleate and potential process disturbances related to LCFA inhibition. From day 172, 2.0 g oleate l^−1^ were added every second day (i.e., pulse feeding) instead of 1.0 g l^−1^ every day. Digesters R1 and R4 received PASS as the only substrate, operating as controls. The PASS and oleate loadings were stopped on day 201, in order to evaluate the residual biogas production associated with the undegraded substrate in batch-mode operating phase (Table 1).

### Process monitoring

Biogas production in the digesters was measured using gas meters, working on the principle of liquid displacement (Belach Bioteknik, Skogås, Stockholm, Sweden). Biogas formation was automatically recorded at 20-min intervals and cumulative volume of the biogas between feeding cycles is reported at 20 °C and 1.013 bar. Biogas composition, including methane, carbon dioxide, oxygen, and hydrogen sulfide, was determined weekly during continuous operation of the digesters, using a portable gas analyzer (Biogas Check, Geotech, Chelmsford, UK). The TS and VS content of the effluent were measured once a week according to the Swedish Standard method SS028113.

The pH was measured using a pH meter twice a week (InoLab 7310, WTW, Weilheim, Germany) and volatile fatty acids (VFA), i.e., acetate, propionate, butyrate, isobutyrate, valerate, isovalerate, caproate, and isocaproate, were quantified weekly using a gas chromatograph (6890 Series, Hewlett Packard, USA) according to Jonsson and Boren [23]. Measurements of myristic, palmitic, stearic, and oleic acid concentrations were performed according to a method adapted from Ziels et al. [24]. In short, 1 ml sludge samples were transferred to 10 ml glass vials, followed by 2 ml of hexane:methyl tert-butyl ether solution (1:1 vol. ratio), 2 drops of sulfuric acid (50% vol. ratio), 200 μl of sodium chloride in ultrapure water (250 g l^−1^), and 100 μl of internal standard (1 g l^−1^ pentadecanoic acid in hexane:methyl tert-butyl ether). The solution was vortexed and further suspended for 20 min on an orbital shaker at 250 rpm. The solution was centrifuged (1600 × *g*, 10 min) and the supernatant was separated for further analysis by a gas chromatograph (6890 Series, Hewlett Packard, USA).

### Microbial community analysis

Triplicate samples were retrieved on days 0 (inoculum), 84 (at the end of start-up phase), 128, 171 (during semi-continuous oleate feeding), 186, 200 (during oleate pulse feeding), 205, 221, and 254 (during batch operation) and used for microbial community analysis. DNA extraction from the samples was carried out using the FastDNA spin kit for soil (MP Biomedicals, Santa Ana, CA, USA) and DNA concentrations were quantified by a Qubit 4 Fluorometer (Invitrogen, Thermo Fisher Scientific, Waltham, MA, USA). The 16S rRNA genes were amplified by polymerase chain reaction (PCR), using primer pair 515′F(GTGBCAGCMGCC GCG GTAA)/805R(GAC TAC HVGGG TAT CTA ATC C) for obtaining the bacteria sequence library and 516F(TGY CAG CCG CCG CGG TAA HACCVGC)/915R(GTG CTC CCC CGC CAA TTC CT) for the archaea sequence library [25, 26]. For details of the PCR procedure used for amplification of the 16S rRNA genes, see Müller et al. [27].

The DNA extracts were processed for next-generation amplicon sequencing by Illumina MiSeq technology at the SNP&SEQ Technology Platform of the SciLifeLab in Uppsala, Sweden. Taxonomic profiles were assigned after processing the raw sequencing data by DADA2 software and the rRNA database SILVA, release 132, based on amplicon sequence variants (ASV) [28–30]. The primers and indices were trimmed by Cutadapt to filter N-based sequences [31]. Forward and reverse sequences were cut to lengths 240 and 160 bp for bacteria and 240 and 200 bp for archaea according to their quality profile, with the quality threshold of maxEE = 2 and truncQ = 11. The raw sequencing data are accessible via the National Center for Biotechnology Information database (identification number SRP276649). For determination of the relative contribution of the identified taxa, archaea sequences were omitted from the bacterial sequence library and bacteria sequences from the archaeal sequence library.

Weighted UniFrac principal coordinate analysis (PCoA) was applied to assess the phylogenetic distance of the microbial community among the samples [32]. A co-occurrence network of bacteria and archaea at genus level was constructed using ASV with relative abundance ≥ 3% of total sequences, according to the approach recommended by Williams et al. [33]. Spearman’s rank correlation was calculated between each pair of ASV, and *p*-values were corrected by Benjamini–Hochberg correction for controlling false discovery rate upon multiple comparisons [34]. Highly significant correlations (*p* ≤ 0.001, correlation coefficient ≥ 0.5) are reported in the co-occurrence network. Degree, betweenness, and closeness centrality indices were determined in order to assess the interaction structures in the co-occurrence network, according to Jordán [35]. Statistical analyses were performed by R software [36], using the vegan [37] and network [38] packages.

## Results

### Effect of effluent solids recirculation on anaerobic digestion of PASS (R1 and R4)

Average biogas production in digester R1, which received PASS as the only substrate, was 3900 ± 730 ml day^−1^ (day 0–200), corresponding to specific biogas production of 510 ± 80 ml g^−1^ VS_substrate_ day^−1^ with methane content of 61 ± 2% of biogas. Biogas production from PASS in R4, with effluent recirculation, was slightly higher than in R1 (*t*-test, *p* < 0.01) with an average value of 4300 ± 650 ml day^−1^ during 200 days of operation (specific biogas production: 570 ± 100 ml g^−1^ VS_substrate_ day^−1^; methane content: 60 ± 2% of biogas). As a result of effluent recirculation, TS content in R4 increased from 2.6 ± 0.2% of total weight during the start-up phase to 4.6 ± 0.2% towards the end of the experiment (day 172–200), whereas the relative VS content declined from 56 ± 2 to 43 ± 1% of TS (Table 2). The pH of sludge in R4 was slightly, but significantly, higher than in R1 (7.6 ± 0.2 and 7.4 ± 0.1, respectively; *t* test, *p* < 0.01). The VFA and LCFA concentrations were below the quantification limits of 40 and 80 mg l^−1^, respectively, throughout the experiment. Accordingly, effluent recirculation led to higher average daily biogas production by ~ 10%, a higher TS content with a larger proportion of inorganic fractions (i.e., lower VS content), and higher pH by ~ 0.2 units during the PASS digestion.

**Table 2:** Parameters monitored during operation of the laboratory stirred-tank anaerobic digesters (R1–R6)

	Days^a^	R1	R2	R3	R4	R5	R6
Average specific biogas production (ml g^−1^VS_substrate_ day^−1^)^b^	0–84	510 ± 95	590 ± 100	570 ± 110	610 ± 100	610 ± 90	570 ± 90
	85–171	510 ± 75	590 ± 80	590 ± 80	525 ± 85	940 ± 310	950 ± 310
	172–200	515 ± 65	745 ± 400	821 ± 480	590 ± 80	1540 ± 175	1315 ± 310
Cumulative residual biogas (ml)^c^	201–255	16,340	108,930	85,240	25,280	73,360	97,130
Cumulative residual biogas from oleate (ml)^d^	201–255	0	92,590	68,900	0	48,080	71,850
Methane (% of biogas)	0–84	61 ± 2	60 ± 2	64 ± 1	60 ± 1	61 ± 1	64 ± 1
	85–171	61 ± 1	62 ± 3	64 ± 1	60 ± 1	61 ± 2	66 ± 1
	172–200	61 ± 2	63 ± 1	65 ± 1	60 ± 2	64 ± 3	66 ± 2
Gaseous H_2_S (ppm)	0–84	< 20	< 20	50 ± 35	< 20	< 20	50 ± 30
	85–171	< 20	< 20	190 ± 80	< 20	< 20	70 ± 20
	172–200	< 20	< 20	320 ± 200	< 20	< 20	150 ± 15
pH	0–200	7.4 ± 0.1	7.4 ± 0.1	7.5 ± 0.1	7.6 ± 0.2	7.6 ± 0.2	7.7 ± 0.2
Total solids (% of total weight)	0–84	2.1 ± 0.3	1.9 ± 0.3	2.4 ± 0.1	2.6 ± 0.2	2.4 ± 0.5	2.8 ± 0.4
	85–171	2.0 ± 0.4	2.3 ± 0.4	2.9 ± 0.4	3.5 ± 0.7	3.6 ± 0.4	4.1 ± 0.7
	172–200	2.3 ± 0.4	3.5 ± 0.2	3.9 ± 0.8	4.6 ± 0.2	5.0 ± 0.5	5.5 ± 0.3
Volatile solids (% of TS)	0–84	56 ± 2	58 ± 2	53 ± 2	56 ± 2	56 ± 2	53 ± 3
	85–171	53 ± 2	54 ± 3	50 ± 2	47 ± 4	48 ± 3	45 ± 3
	172–200	50 ± 1	61 ± 5	61 ± 6	43 ± 1	48 ± 2	43 ± 2

^a^Day 0–84: start-up phase; day 85: start of semi-continuous oleate feeding to R2, R3, R5, and R6; day 172: start of oleate pulse feeding; day 201: end of PASS and oleate feeding

^b^Calculation of specific biogas production is based on the influent VS content of fresh substrate, does not include the recirculated VS

^c^Cumulative biogas production between days 201 and 255 after the PASS and oleate feeding was stopped

^d^Cumulative residual biogas production from oleate calculated by subtracting the cumulative biogas production in control digester, R1, from the values obtained for oleate-amended R2 and R3 and subtracting the cumulative biogas production of control digester with effluent recirculation, R4, from the values obtained for R5 and R6

### Effect of effluent solids recirculation on oleate conversion to biogas (R2 and R5)

**Fig. 1:**
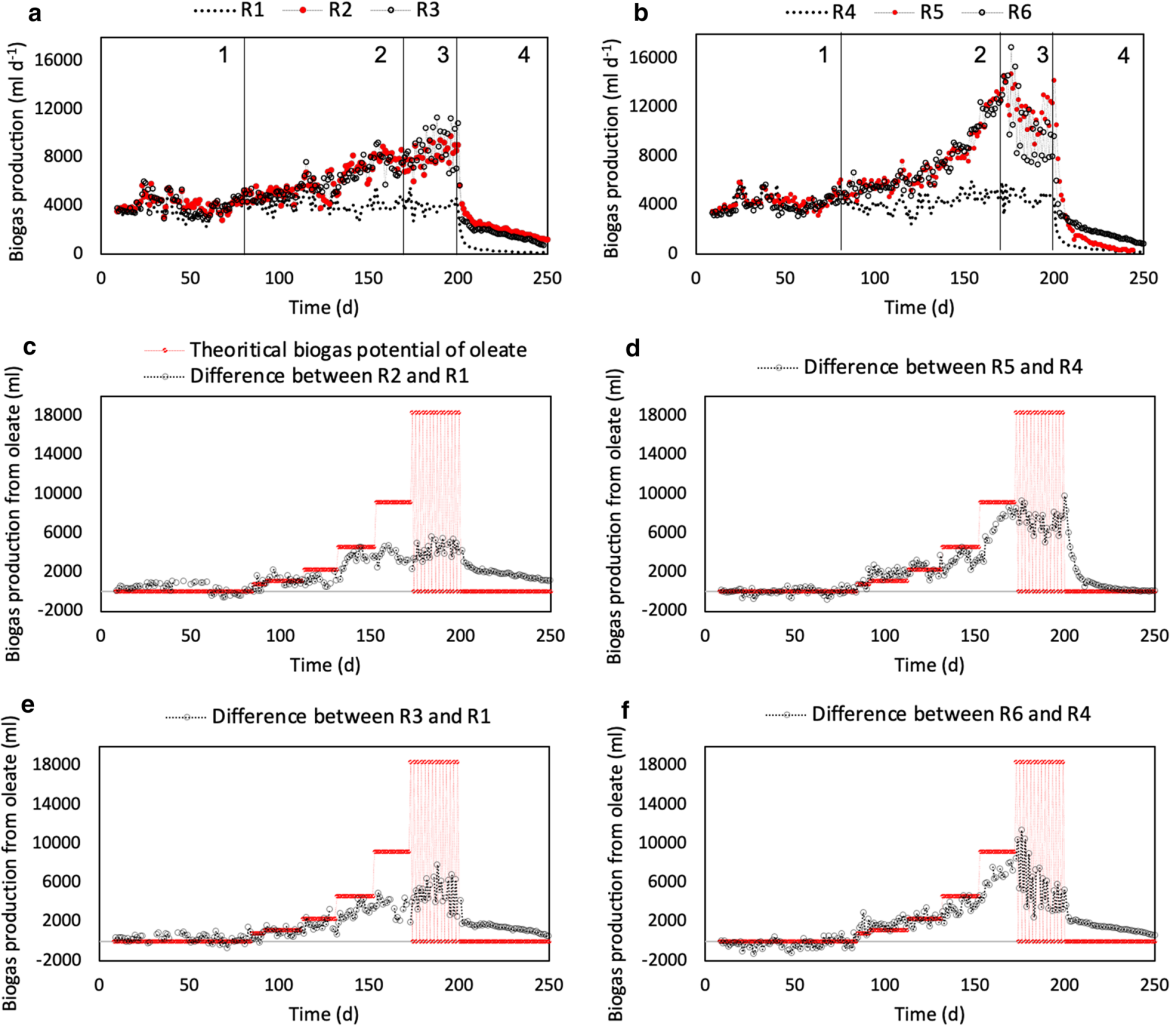
Daily biogas production in digesters R1–R6 (**a**, **b**). (1) Start-up phase, (2) semi-continuous oleate feeding, (3) oleate pulse feeding, and (4) end of PASS and oleate feeding. Daily biogas production associated with oleate degradation, based on differences in daily biogas production between oleate-amended and control digesters R2 and R1 (**c**), R5 and R4 (**d**), R3 and R1 (**e**), and R6 and R4 (**f**). Theoretical biogas production (red line) was calculated as 1532 ml from 1 g oleate based on the Buswell equation [39]

After a stepwise increase in the oleate loading rate to 0.5 g l^−1^ day^−1^ in R2, the volumetric biogas production from oleate reached an average value of 3500 ± 600 ml day^−1^ between days 131 and 151 (Fig. 1c). During this period, average specific biogas production of 1170 ± 210 ml g^−1^ oleate day^−1^ was obtained, corresponding to 76% of the theoretical biogas potential (1532 ml from 1 g oleate, based on the Buswell equation; [39]). However, further elevation of the oleate loading rate to 1.0 g l^−1^ day^−1^ between days 152 and 171 did not result in higher biogas production and yielded average specific biogas production of only 600 ± 120 ml g^−1^ oleate day^−1^ (39% of the theoretical biogas potential). The subsequent pulse feeding of oleate resulted in a minor, yet significant, increase in specific biogas production to 700 ± 140 ml g^−1^ oleate day^−1^ between days 172 and 200 (*t* test, *p* < 0.05). It can therefore be inferred that oleate conversion was limited in R2 at loading rates higher than 0.5 g l^−1^ day^−1^, yielding ~ 39–46% of the theoretical biogas potential at 1.0 g oleate l^−1^ day^−1^.

In contrast to R2, biogas production from oleate in R5 steadily increased from 3400 ml on day 152 to 8100 ml on day 171 after elevating the oleate loading rate from 0.5 to 1.0 g l^−1^ day^−1^ (Fig. 1d). Average volumetric biogas production from oleate in R5 was 6400 ± 1700 ml day^−1^ between days 152 and 171, which corresponded to specific biogas production of 1120 ± 270 ml g^−1^ oleate day^−1^ (73% of the theoretical potential). Further pulse feeding of 2.0 g oleate l^−1^ every second day resulted in a temporary decline in daily biogas production but did not have any significant effect (*t* test, *p* > 0.05) on the overall efficiency of oleate conversion in R5 (specific biogas production of 1220 ± 190 ml g^−1^ oleate day^−1^ between days 172 and 200). The pH of sludge in R2 and R5 was relatively constant throughout the experiment, with slightly higher pH in R5 compared with R2 (7.6 ± 0.2 and 7.4 ± 0.1, respectively) and VFA concentrations remained below 60 mg l^−1^ throughout the experiment (mainly acetate; data not shown).

The LCFA identified in effluents of R2 and R5 were palmitic, stearic, and oleic acids, whereas myristic acid was not detected (Additional file 1: Fig. S1). In R2 effluent, total concentration of specific LCFA (i.e., sum of palmitic, stearic, and oleic acids) increased from < 80 mg l^−1^ on day 84 to 1500 ± 370 mg l^−1^ on day 151 at an oleate loading rate of 0.5 g l^−1^ day^−1^ (17% palmitic acid, 41% stearic acid, 42% oleic acid). The specific LCFA in effluent of R5 also increased, but to a lower extent, from < 80 to 580 ± 100 mg l^−1^ during the same period (17% palmitic acid, 41% stearic acid, 42% oleic acid). Following an increase in oleate loading rate from 0.5 to 1.0 g l^−1^ day^−1^ and oleate pulse feeding, effluent concentration of LCFA in R5 went up to ~ 2000 mg l^−1^, followed by a decline to < 80 mg l^−1^ after cessation of oleate and substrate feeding on day 200. At oleate loading of 1.0 g l^−1^ day^−1^ in R2, concentration of specific LCFA remained at a relatively constant level (~ 1500 mg l^−1^), followed by a gradual decline towards the end of the experiment (Additional file 1: Fig. S1).

In summary, comparison of the performance of digesters R2 and R5 during semi-continuous (days 85–171) and pulse feeding (days 172–200) of oleate indicated that effluent recirculation led to higher LCFA degradation capacity and more efficient conversion of oleate to biogas at loading rates up to 1.0 g l^−1^ day^−1^. Furthermore, the rate of daily biogas production was faster in R5 compared with R2 at higher loads of oleate, based on evolution of biogas between feeding cycles (Additional file 1: Fig. S2). In line with these observations, cumulative residual biogas production from oleate in R5 was approximately half of that in R2 during the batch-mode operation (days 201–255) (Table 2), suggesting more efficient conversion of oleate to biogas in R5 with effluent recirculation during semi-continuous and pulse feeding.

### Effect of sulfide on oleate conversion to biogas (R3 and R6)

For the digesters with sulfide added (R3 and R6), the major difference compared with the other digesters was observed during the pulse feeding of oleate, where biogas production between the feeding intervals showed larger variations (Fig. 1e, f). Average specific biogas production in this period was significantly (*t* test, *p* < 0.01) higher for sulfide-amended R3 than for R2, and the amount of biogas produced from residual oleate during the batch phase was lower, implying a higher degree of oleate conversion during the preceding steps (Table 2). However, the opposite pattern was observed when comparing R6–R5, where sulfide-amended R6 showed a decreasing trend in biogas production and produced a lower amount of biogas from oleate during the oleate pulse feeding and batch-mode operation (Fig. 1f; Table 2). It is therefore evident that sulfide addition allowed for more efficient conversion of oleate in the digester without effluent recirculation (R3), while it had a negative influence on oleate conversion to biogas in the digester with effluent recirculation (R6).

Nonetheless, oleate conversion in the sulfide-amended digester with effluent recirculation (R6) was more efficient at loading rates up to 1.0 g oleate l^−1^ day^−1^ than in the sulfide-amended digester without effluent recirculation (R3), and the rate of daily biogas production was faster (Fig. 1e, f; Additional file 1: Fig. S2). In line with these results, the highest level of LCFA accumulation was observed for samples collected from R3 after the oleate pulse feeding, increasing from < 80 to 7400 ± 1600 mg l^−1^ on day 199 (77% palmitic acid, 12% stearic acid, 11% oleic acid). The total concentration of specific LCFA in R6 reached a maximum value of 2000 ± 1600 mg l^−1^ on day 171 after increasing the oleate loading rate to 1.0 g l^−1^ day^−1^ (34% palmitic acid, 33% stearic acid, 33% oleic acid). The pH in R3 and R6 remained relatively constant throughout the experiment, with higher pH in R6 compared with R3 (7.7 ± 0.2 and 7.5 ± 0.1, respectively). The VFA concentrations remained below 60 mg l^−1^ throughout the experiment (mainly acetate; data not shown). Accordingly, the assessment of R3 and R6 performance also indicated that effluent recirculation led to more efficient conversion of oleate to biogas at loading rates up to 1.0 g l^−1^ day^−1^ at elevated sulfide level in the digester.

### Bacterial community dynamics

**Fig. 2:**
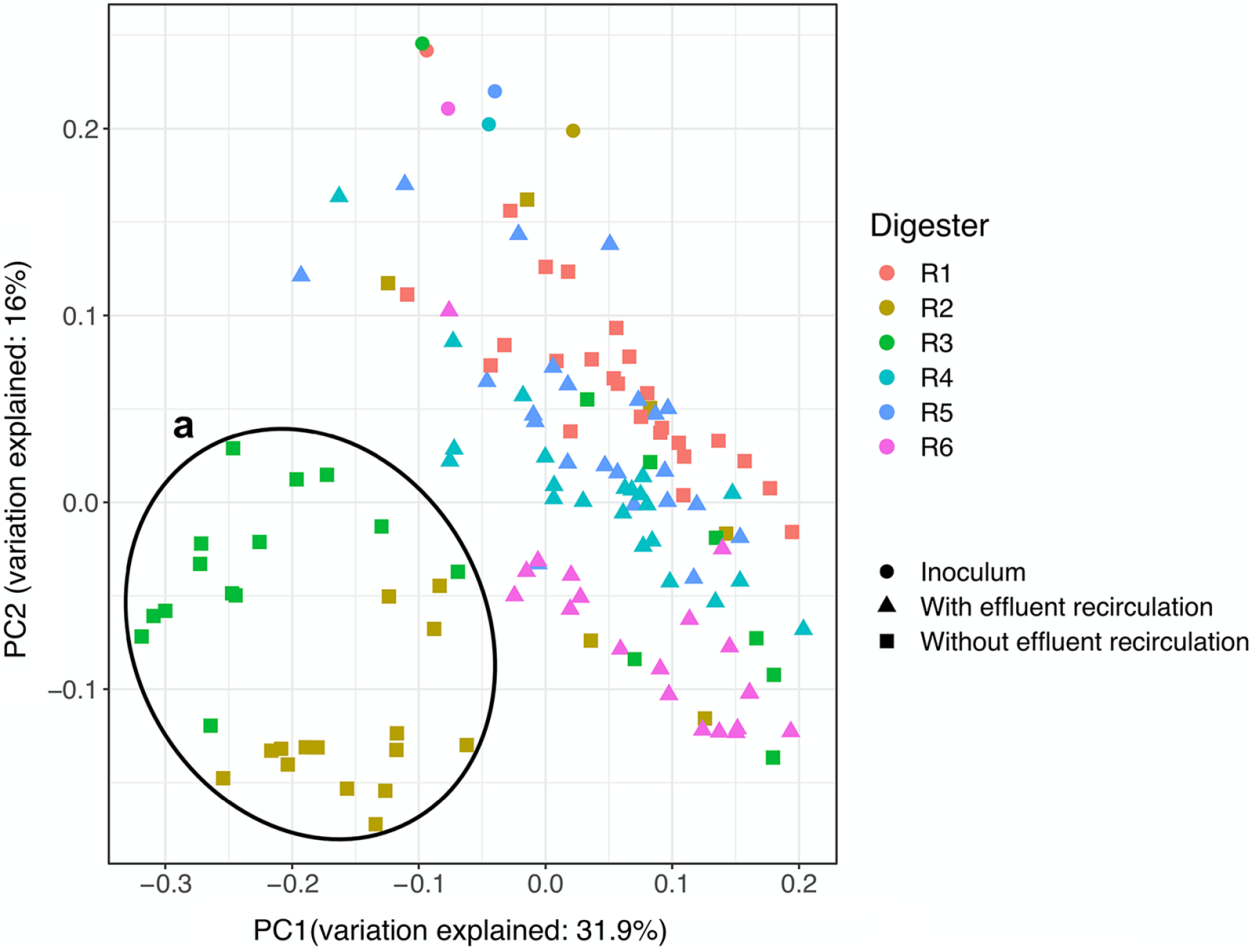
Phylogenetic distance of the bacterial community determined by weighted UniFrac principal coordinate (PC) analysis of the ASV read counts. **a** Samples collected from digesters R2 and R3 after initiating oleate pulse feeding

After quality trim and chimera control of the raw data from sequencing of bacterial 16S rRNA genes, 2075 to 305943 sequence reads per sample were obtained (25th and 75th percentiles of 12353 and 26707 sequence reads per sample, respectively). Weighted UniFrac PCoA of the ASV read counts revealed that the initial bacterial community structure in the digesters (i.e., inoculum) gradually diverged depending on whether or not effluent recirculation, oleate addition or sulfide addition was applied (Fig. 2). The bacterial community in digesters R2 and R3 after oleate pulse feeding showed the highest degree of phylogenetic dissimilarity to other digesters, forming separate groupings in the PCoA plot. In contrast, samples from oleate-supplemented digesters with effluent recirculation (R5 and R6) were closer to those collected from control digesters (R1 and R4). It is therefore apparent that the pulse feeding of oleate altered the bacterial community structure primarily in the digesters without effluent recirculation. Moreover, the separation of R2 and R5 samples from their corresponding sulfide-amended digesters (R3 and R6, respectively) in the PCoA plot signifies the phylogenic distances in the bacterial community related to addition of sulfide (Fig. 2).

**Fig. 3:**
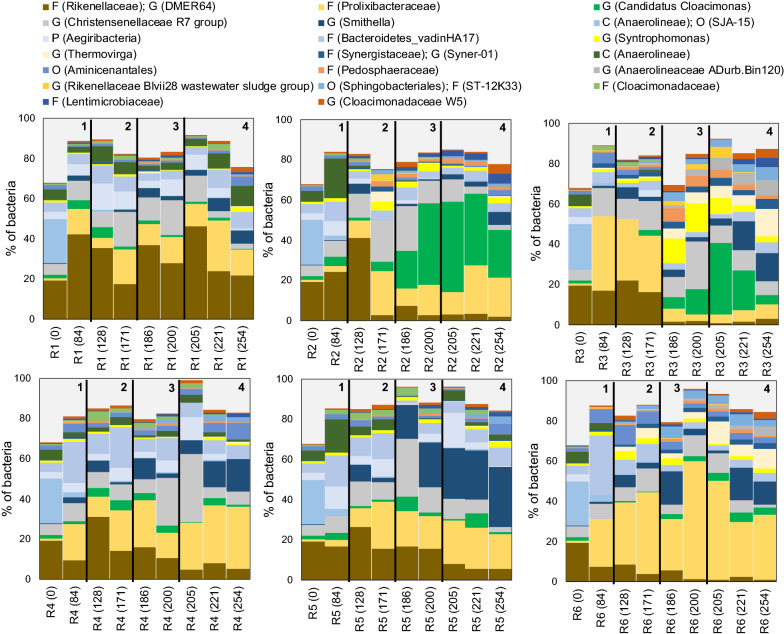
Relative abundance of bacterial 16S rRNA genes at genus (G) level based on the average ASV reads from triplicate samples, collected on different days. Where genus name could not be assigned to the sequences, the closest classified taxonomic level is presented; phylum (P), class (C), order (O), family (F). Bacteria with relative abundance of ≥ 3% of total bacteria on at least one sampling occasion are depicted. (1) Start-up phase, (2) semi-continuous oleate feeding, (3) oleate pulse feeding, and (4) end of PASS and oleate feeding

The ASV assigned to the phyla Chloroflexi, Bacteroidetes, and Firmicutes had the highest relative abundances in the inoculum (31 ± 4.4, 26 ± 2.4, and 15 ± 3.7% of bacteria, respectively). After the start-up phase, Bacteroidetes and Firmicutes remained dominant in the digesters, with varying relative abundances, whereas Chloroflexi substantially decreased in relative abundance in all digesters over time (Additional file 1: Fig. S3). Other bacterial phyla which were present in high relative abundance on at least one sampling occasion included Cloacimonetes (< 2.0 to 45 ± 6.0% of bacteria), Proteobacteria (< 2.0 to 31 ± 5.2% of bacteria), Synergistetes (< 2.0 to 19 ± 7.4% of bacteria), Aegiribacteria (< 2.0 to 17 ± 10% of bacteria), and Acidobacteria (< 2.0 to 9.8 ± 2.0% of bacteria). The genus DMER64 (family Rikenellaceae) was the dominant member of the phylum Bacteroidetes and occurred with lower relative abundances in the digesters with effluent recirculation (Fig. 3). An increase in oleate loading rate and further oleate pulse feeding resulted in a decline in relative abundance of this genus, particularly in R2 and R3 (Fig. 3). In contrast, the family Prolixibacteraceae, also belonging to the phylum Bacteroidetes, prevailed in the digesters with effluent recirculation throughout the experiment, with highest relative abundances at the elevated sulfide level of R6 (up to 59 ± 3% of bacteria).

Major differences in the bacterial community were observed subsequent to pulse feeding of oleate to the digesters (Fig. 3). In particular, the relative abundance of the genus *Candidatus Cloacimonas* (phylum Cloacimonetes; family Cloacimonadaceae) in R2 and R3 increased from 4.6 ± 1.0 to 45 ± 6 and from 2.8 ± 1.0 to 36 ± 4.4% of bacteria, respectively, with a subsequent decline during batch-mode operation (Fig. 3). The relative abundance of this genus did not change in the digesters with effluent recirculation (R5 and R6) or in control digesters (R1 and R4), where it remained at < 7.0% of bacteria. The genus *Smithella* (phylum Proteobacteria; family Syntrophaceae) prevailed in R5 after oleate pulse feeding, with a gradual increase from < 2.0 to 30 ± 5.2% of bacteria from day 172 onward (Fig. 3). The relative abundance of *Smithella* also increased in the control digester with effluent recirculation (R4), to 16 ± 4.3% of bacteria, and in sulfide-amended digesters during the batch-mode operation (R3 and R6), to 14 ± 0.5 and 16 ± 6.0% of bacteria, respectively (Fig. 3). Together, these observations suggest that oleate pulse feeding promoted relative abundance of *Candidatus Cloacimonas* in the digesters without effluent recirculation, while occurrence of *Smithella* was promoted mainly in the digesters with effluent recirculation and/or sulfide addition.

The relative abundance of the genus *Syntrophomonas* (phylum Firmicutes; family Syntrophomonadaceae) substantially increased, from < 2.0 to 14 ± 4.7% of bacteria, during pulse feeding of oleate to sulfide-amended R3, but steadily declined after the feeding was stopped on day 200 (Fig. 3). As the relative abundance of *Syntrophomonas* remained at < 4.0% of bacteria in all digesters except R3, oleate pulse feeding and a higher sulfide level apparently contributed to an increase in the relative abundance of this genus in the digester without effluent recirculation. A characteristic of the bacterial community in the sulfide-amended digesters, R3 and R6, was prevalence of the genus *Thermovirga* (phylum Synergistetes; family Synergistaceae), with increasing relative abundance from < 2.0 to 14 ± 7.0 and 12 ± 4.6% of bacteria, respectively, from day 172 onward (Fig. 3).

### Archaeal community dynamics

**Fig. 4:**
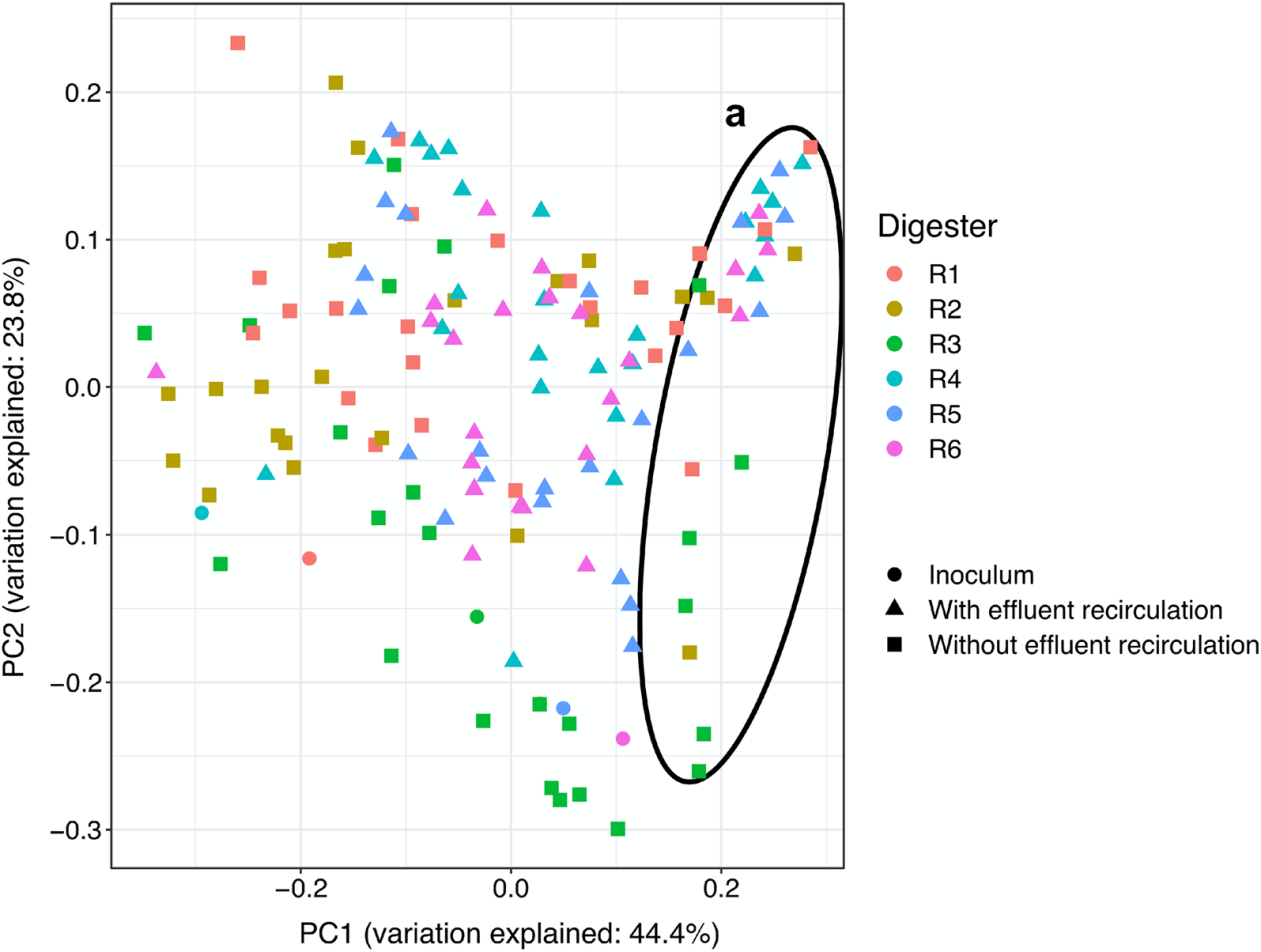
Phylogenetic distance of the archaeal community determined by weighted UniFrac principal coordinate (PC) analysis of archaeal ASV read counts. **a** Samples collected during batch-mode operation

After quality trim and chimera check of the raw data from sequencing of archaeal 16S rRNA genes, 4794 to 248356 sequence reads per sample were acquired (25th and 75th percentiles of 13893 and 29463 sequence reads per sample, respectively). The phylogenetic distance of the archaeal community from different digesters was less pronounced in the UniFrac PCoA plot than observed for bacteria (Figs. 2, 4). Nevertheless, a divergence from initial archaeal community structure (i.e., inoculum) was evident, where samples from R2 and R3, which received oleate without effluent recirculation, tended to position at a longer distance from the other samples (Fig. 4). Samples collected during batch-mode operation of the digesters positioned closer to each other, suggesting that the degree of dissimilarity among the archaeal community structures was reduced upon cessation of feeding.

**Fig. 5:**
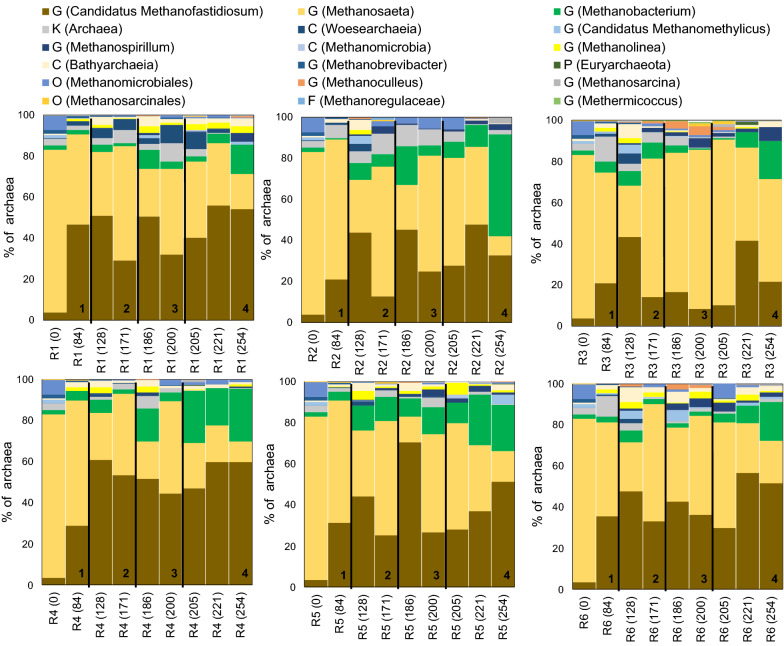
Relative abundance of archaeal 16S rRNA genes at genus (G) level based on the average ASV reads from triplicate samples, collected on different days. Where genus name could not be assigned to the sequences, the closest classified taxonomic level is presented; kingdom (K), phylum (P), class (C), order (O), family (F). (1) Start-up phase, (2) semi-continuous oleate feeding, (3) oleate pulse feeding, and (4) end of PASS and oleate feeding

The archaeal community of the inoculum was dominated by the phylum Euryarchaeota (97 ± 1.6% of archaea), with a minor contribution from Crenarchaeota (2.4 ± 0.7% of archaea). The genus *Methanosaeta* (family Methanosaetaceae) was the dominant member of the Euryarchaeota (79 ± 3.6% of archaea) in the inoculum, with a subsequent decrease in relative abundance over time in all digesters (Fig. 5). The genus *Candidatus Methanofastidiosum* (phylum Euryarchaeota; family Methanofastidiosaceae) had relative abundance of < 2.0% of archaea in the inoculum, while it successively prevailed in all digesters with relative abundance up to 71 ± 11% of archaea (Fig. 5). An overall increasing trend in relative abundance of *Methanobacterium* (phylum Euryarchaeota; family Methanobacteriaceae) was observed in all digesters, with greater occurrence in the oleate-amended digesters towards the end of batch-mode operation (Fig. 5). Among less abundant species, unidentified members of the order Methanomicrobiales (phylum Euryarchaeota) decreased in relative abundance from 6.9 ± 3.4 in the inoculum to < 2.0% of archaea during the start-up phase (day 0–84). Oleate addition led to re-occurrence of Methanomicrobiales, particularly in R2 with relative abundance of 5.8 ± 0.5% of archaea at the end of the semi-continuous and pulse feeding, following a decline to < 2.0% of archaea upon batch-mode operation. The relative abundance of the genus *Methanoculleus* (phylum Euryarchaeota; family Methanomicrobiaceae) was particularly high in sulfide-amended digesters after initiation of oleate pulse feeding, with the maximum value observed in R3 on day 200 (4.1 ± 2.0% of archaea).

### Co-occurrence of bacteria and archaea

**Fig. 6:**
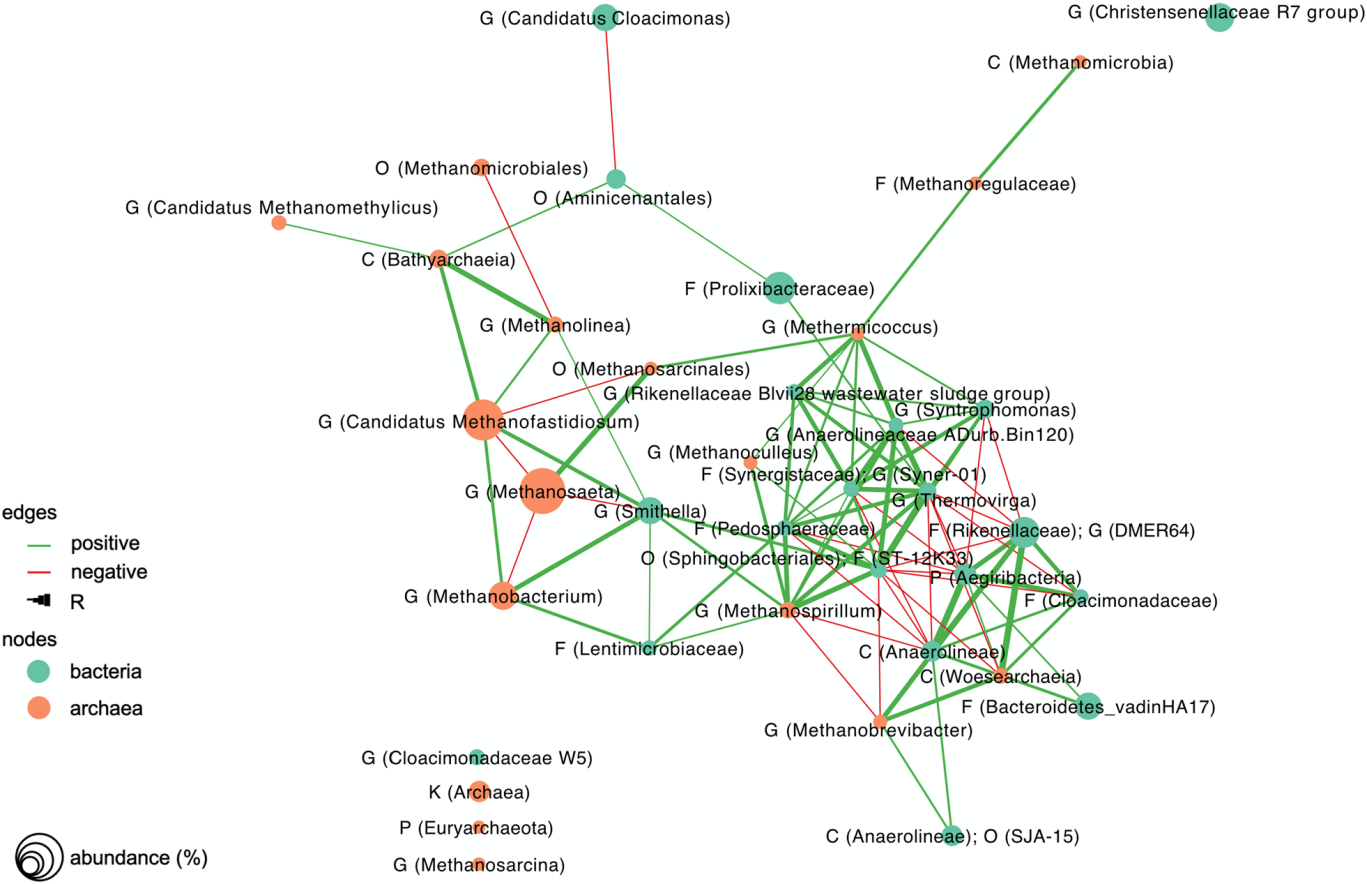
Co-occurrence network analysis based on correlation of the relative abundance of ASV reads for bacterial and archaeal profiles at genus or the closest classified taxonomic level. Bacterial and archaeal groups are shown by nodes in green and orange, respectively. Each edge represents significant correlations between a pair of nodes (*p* ≤ 0.001), where positive and negative correlations are colored in green and red, respectively. The thickness of the edge is proportional to the R value of the correlation (*R* ≥ 0.5)

The co-occurrence network included 21 bacteria and 12 archaea with significant correlations. Three subclusters were identified in network analysis, including two bacterial clusters and the most dominant archaea as a separate group at the edge of the co-occurrence network (Fig. 6). The two bacterial groups were negatively correlated to each other, while the relative abundance of taxa within each group was positively correlated (Fig. 6). Within the archaeal group, the relative abundance of *Methanosaeta* was negatively correlated to that of *Candidatus Methanofastidiosum* and *Methanobacterium*. Considering the high values of network statistics used in quantitative representation of keystone species (i.e., degree, betweenness, and closeness centrality [35]), *Smithella* and *Thermovirga* stood out as potentially influential genera (Additional file 1: Table S1). *Smithella* had the highest betweenness centrality, bridging the bacterial clusters to the cluster of dominant archaea, where its relative abundance was positively correlated to *Candidatus Methanofastidiosum* and *Methanobacterium* (and negatively to *Methanosaeta*). *Thermovirga* had the highest number of neighbors (i.e., degree centrality), and the shortest average distance to other nodes in the network (i.e., closeness centrality). A positive correlation was also observed between low-abundance archaea, *Methanoculleus*, *Methanomicoccus*, *Methanoregulaceae*, and *Methanomicrobia,* with more closeness to the bacterial aggregates in the network. *Candidatus Cloacimonas* was identified as the genus with the lowest degree, betweenness, and closeness centrality among the bacteria (Additional file 1: Table S1).

## Discussion

### Influence of effluent solids recirculation

Effluent solids recirculation enabled more efficient conversion of oleate to biogas (78% of theoretical biogas potential) at loading rates up to 1.0 g l^−1^ day^−1^, compared with the digesters without effluent recirculation (38% of theoretical biogas potential). Effluent solids recirculation also resulted in establishment of a microbial community in the oleate-amended digesters, with closer phylogenetic distance to the control digesters compared with the digesters without effluent recirculation. Defining resistance of the microbial community as the extent of community alternation upon disturbances [40], it can be inferred that effluent recirculation enhanced microbial community resistance to the high loads of LCFA in this study.

Enhanced oleate conversion capacity might relate to longer retention of solids-bound oleate in the digesters, which could also allow greater cell growth in fatty acid-degrading consortia with slow growth rates (e.g., 0.10–0.19 day^−1^ for propionate and butyrate-degrading consortia, 0.7–2.2 day^−1^ for LCFA-degrading consortia [7, 41]). In line with this suggestion, we observed that daily biogas production after feeding PASS and oleate was faster at higher loads of oleate in R5 and R6 compared with oleate-amended R2 and R3. This points to enhanced kinetic capacity of the microbial biomass for substrate degradation and turnover of intermediate degradation products, such as VFA, to biogas [42]. Furthermore, increased solids content due to effluent recirculation may facilitate dispersion and availability of oleate for microorganisms by providing a larger surface area for adsorption, which could also mediate potential inhibitory effects of LCFA, as previously shown after supplying additive particles (e.g., bentonite; [43]).

Two mechanisms have been proposed for initial β-oxidation of unsaturated LCFA such as oleate: (i) Sequential biohydrogenation of olefinic bonds (e.g., oleate to stearic acid), followed by β-oxidation to the corresponding saturated LCFA (e.g., stearic acid to palmitic acid); and/or (ii) formation of unsaturated intermediates (e.g., oleate to palmitoleic acid), prior to double-bond biohydrogenation [44–46]. Simultaneous occurrences of stearic and palmitic acids after introducing oleate to R2, R3, R5, and R6 imply that biohydrogenation and β-oxidation of oleate primarily occurred sequentially through separate routes (Additional File 1: Fig. S1). It has frequently been observed that palmitic acid accumulates as the major intermediate product in digesters fed by oleate, suggesting that the kinetics of palmitic acid degradation are the rate-limiting factor for oleate β-oxidation [47]. Similarly, we observed that the concentration of palmitic acid increased in the digesters upon semi-continuous and pulse feeding of 1.0 g oleate l^−1^ day^−1^, yet at a generally lower level when effluent solids recirculation was implemented (Additional File 1: Fig. S1). Therefore, effluent recirculation may have contributed to a higher degree of palmitic acid conversion during oleate β-oxidation and, thus, to greater oleate conversion capacity.

### Influence of oleate feeding frequency

Oleate feeding frequency (semi-continuous versus pulse feeding) appeared to have a substantial impact on the biogas process, as abrupt alterations in the bacterial community composition and daily kinetics of biogas production were observed after pulse feeding (Additional File 1: Fig. S2). Following oleate pulse feeding, the genus *Candidatus Cloacimonas* (phylum Cloacimonetes) prevailed in R2 and was associated with a higher degree of oleate conversion to biogas. *Candidatus Cloacimonas* belongs to the phylum Cloacimonetes, which has been commonly observed in anaerobic environments, including full-scale biogas plants [48]. The syntrophic lifestyle of the species of *Candidatus Cloacimonas* was previously suggested based on identification of genes related to syntrophic propionate oxidation in the presence of hydrogen-utilizing microorganisms [49]. In addition, the occurrence of members of phylum Cloacimonetes (e.g., Cloacimonadales W27, *Cloacidonadaceae W5*, and *Candidatus Cloacimonas*) during anaerobic lipids and LCFA degradation in different anaerobic environments has been used as an argument for their potential involvement in lipid degradation [16, 50, 51], which is in line with our observation on the prevalence of *Candidatus Cloacimonas* in R2 following oleate pulse feeding. Nevertheless, *Candidatus Cloacimonas* had the lowest degree, betweenness, and closeness centrality among the bacteria in the co-occurrence network, suggesting that this genus had limited interactions with other microorganisms [35] and, therefore, its contribution to the integrity and functions of the microbial community is possibly marginal.

Bacterial community response to the oleate pulse feeding was different in R5, with effluent recirculation, where *Smithella* prevailed instead of *Candidatus Cloacimonas* as compared to R2. *Smithella* is able to oxidize fatty acids (e.g., propionate and butyrate), *n*-alkanes (e.g., C_9_–C_12_), and an association of *Smithella* to LCFA conversion in PASS digesters has been reported [16, 52, 53]. *Smithella* is also able to dismutate propionate to butyrate and acetate, and it has low sensitivity to high hydrogen partial pressure, e.g., during syntrophic propionate oxidation [52, 54]. It may therefore be argued that these features of *Smithella* might have provided a growth advantage over hydrogen-sensitive organic acid oxidizers during pulse feeding of oleate to R5, when hydrogen formation via β-oxidation likely occurred at a higher rate compared with R2 (i.e., the amount and kinetics of biogas produced from oleate in R5 were higher than in R2). It is also noteworthy that the relative abundance of *Smithella* increased in the control digester R4, suggesting a potential positive contribution of effluent solids recirculation to growth of this genus. High betweenness centrality of *Smithella*, together with significant correlations of its relative abundance to the methanogens, suggest a central role of this genus in supporting interspecies interactions, which was apparently promoted by applying effluent recirculation to the PASS digesters.

### Influence of elevated sulfide level

Oleate conversion to biogas showed lower efficiency at elevated sulfide level in the digesters with effluent recirculation (R6 compared with R5), whereas higher average biogas production from oleate was achieved at elevated sulfide level in the digesters without effluent recirculation, particularly upon pulse feeding (R3 compared with R2). During oleate pulse feeding in R3, the relative abundance of β-oxidizing *Syntrophomonas* species increased substantially, in parallel with the effluent concentration of palmitic acid, which was at the highest level measured in the samples (Additional file 1: Fig. S1)*.* Thus, an increase in relative abundance of LCFA-degrading *Syntrophomonas* apparently promoted the initial step of oleate β-oxidation to palmitic acid in R3, whereas further palmitic acid conversion was still limited. Analysis of the archaeal community indicated presence of *Candidatus Methanofastidiosum*, *Methanosaeta*, and *Methanobacterium* as the most abundant genera in all digesters. Syntrophic partnership of hydrogenotrophic *Methanobacterium* with *Syntrophomonas* has been observed in co-culture (*S. zehnderi* and *M. formicicum*) and a potential syntrophic association between these microorganisms has been suggested in municipal sludge digesters [16, 55]. However, the increase in relative abundance of *Syntrophomonas* in R3 was associated with a decline in the relative abundance of *Methanobacterium* and instead a concomitant increase in the relative abundance of *Methanoculleus* (see Additional file 1: Fig. S4).

*Methanoculleus* is frequently reported as a syntrophic partner of acetate-oxidizing bacteria (reviewed by Westerholm et al. [56]). Syntrophic association of *Syntrophomonas*, *Methanobacterium*, and *Methanoculleus* during β-oxidation in anaerobic cultures acclimatized to a mixture of LCFA (oleate, stearate, palmitate, and myristate) and in propionate-oxidizing chemostats has also been observed [57, 58]. High affinity of *Methanoculleus* for hydrogen, together with its relatively low threshold value for hydrogen partial pressure, have been pointed out as growth advantages of this genus under environmental stresses such as high ammonia levels and micronutrient deficiency [59]. Accordingly, *Methanoculleus* could potentially outcompete *Methanobacterium* for hydrogen at high oleate and sulfide levels in R3 (both regarded as environmental stresses), supporting the growth of syntrophic, β-oxidizing *Syntrophomonas* and higher conversion efficiency of oleate.

The lower biogas production at elevated sulfide level in the digesters with effluent recirculation (R6 compared with R5) could not be attributed to differences in the archaeal community structure, which may be presumed due to inhibition by sulfide. Differences were instead evident for the bacterial community, mainly related to lower relative abundance of *Smithella* in sulfide-amended R6 compared with R5. It is unlikely that the lower relative abundance of *Smithella* in R6 was related to sulfide toxicity, as *Smithella* commonly occurs in S-rich environments (e.g., under sulfate-reducing conditions; [60]) and its resistance to sulfide inhibition has previously been demonstrated [61]. Nevertheless, in the digester combining oleate pulse feeding, increased sulfide level, and effluent recirculation, *Smithella* occurred in lower relative abundances. Based on co-occurrence network analysis, *Smithella* is likely a central species in supporting the interspecies interactions between archaea and bacteria. Thus, it can be inferred that a lower abundance of this genus at elevated sulfide level of R6 might have contributed to lower conversion efficiency of oleate via syntrophic interactions.

A distinct characteristic of the bacterial community in the digesters that received sulfide (R3 and R6) was prevalence of the genus *Thermovirga* (phylum Synergistetes; family Synergistaceae). The known species of this genus, *Thermovirga lienii*, is able to utilize proteinous compounds and reduce cysteine and elemental S to hydrogen sulfide, while fatty acids such as acetate and propionate are not utilized by this species [62]. As sulfide addition is expected to result in formation of elemental S in the digester due to oxidation by influent Fe(III) content of PASS [19, 63], availability of elemental S as an electron acceptor might have contributed to an increase in the relative abundance of S-reducing bacteria such as *Thermovirga*. *Thermovirga* has been suggested as the main protein degrader during anaerobic digestion of a substrate rich in casein, and an increase in the relative abundance of this genus upon sulfide addition to PASS digesters has been reported previously [19, 64]. The highest values of degree and closeness centrality of *Thermovirga* among the bacteria suggest that diverse trophic groups in the anaerobic food chain may be supported by activities of this genus, potentially related to protein fermentation.

## Conclusions

This study showed that effluent solids recirculation to PASS digesters enhances microbial LCFA degradation capacity. Effluent solids recirculation promoted occurrence of the hydrogen-producing, fatty acid-degrading *Smithella*, which likely acted as a keystone species for interactions between bacteria and methanogens. The LCFA pulse feeding and sulfide level had varying impacts on LCFA conversion to biogas and microbial community structure in digesters with and without effluent solids recirculation. A negative effect on oleate conversion to biogas was observed after LCFA pulse feeding at an elevated sulfide level when effluent solids recirculation was applied. However, the LCFA pulse feeding at elevated sulfide level led to prevalence of LCFA-degrading *Syntrophomonas* as well as a more efficient oleate conversion to palmitic acid and biogas in the digester without effluent recirculation. Based on the overall outcomes in this study, application of effluent solids recirculation enables conversion of higher loads of LCFA to biogas, which may provide possibilities for co-digestion of larger amounts of waste lipids together with PASS.

## Supplementary Information

**Additional file 1: Table S1.** (supporting results) Co-occurrence network statistics. **Figure S1.** Concentrations of specific LCFA. **Figure S2** Daily biogas production kinetics. **Figure S3.** Relative abundances of 16S rRNA genes of bacterial phyla. **Figure S4.** Relative abundance of 16S rRNA genes associated to genera *Methanoculleus*, *Methanobacterium*, and *Syntrophomonas* in R3.

## Data Availability

The raw sequencing data are available at the National Center for Biotechnology Information database, under identification number SRP276649.

## Abbreviations

ASV: Amplicon sequence variants
LCFA: Long-chain fatty acids
PASS: Primary and activated sewage sludge
PCoA: Principal coordinate analysis
PCR: Polymerase chain reaction
TS: Total solids
VFA: Volatile fatty acids
VS: Volatile solids
WWTP: Wastewater treatment plant
