# Supplementary material for: Effluent solids recirculation to municipal sludge digesters enhances long-chain fatty acids degradation capacity
Source: Biotechnol Biofuels. 2021 Mar 4;14:56. doi: 10.1186/s13068-021-01913-1 (PMC7934545; doi:10.1186/s13068-021-01913-1)
Supplement: Supplementary file 1 — Additional file 1: Table S1. (supporting results) Co-occurrence network statistics. Figure S1. Concentrations of specific LCFA. Figure S2 Daily biogas production kinetics. Figure S3. Relative abundances of 16S rRNA genes of bacterial phyla. Figure S4. Relative abundance of 16S rRNA genes associated to genera Methanoculleus, Methanobacterium, and Syntrophomonas in R3. [file 13068_2021_1913_MOESM1_ESM.docx]

**Additional file 1 (supporting results)**

**Effluent solids recirculation to municipal sludge digesters enhances long-chain fatty acids degradation capacity**

Sepehr Shakeri Yekta^1,2*^, Tong Liu^2,3^, Thuane Mendes Anacleto^4^, Mette Axelsson Bjerg^1,2^, Luka Šafarič^1,2^, Xavier Goux^5^, Anna Karlsson^2,6^, Annika Björn^1,2^, Anna Schnürer^2,3^

*^1^Department of Thematic Studies-Environmental Change, Linköping University, 58183 Linköping, Sweden*

*^2^Biogas Research Center, Linköping University, 58183 Linköping, Sweden*

*^3^Department of Molecular Sciences, Swedish University of Agricultural Sciences, Uppsala BioCenter, 75007 Uppsala, Sweden*

*^4^Post graduate Program in Plant Biotechnology and Bioprocesses,* *Federal University of Rio de Janeiro, Rio de Janeiro, 21941-901, Brazil*

*^5^Environmental Research and Innovation department, Luxembourg Institute of Science and Technology, 4422 Belvaux, Luxembourg*

*^6^Scandinavian Biogas Fuels AB, 11160 Stockholm, Sweden.*

**Correspondence: sepehr.shakeri.yekta@liu.se; Tel.: +46-13-282-294*

**Table S1 Co-occurrence network statistics, including values of Degree, Betweenness, and Closeness centrality indices for 21 bacteria and 12 archaea with significant correlations. Phylum (P), class (C), order (O), family (F), and genus (G).**

| **Rank** | **Degree centrality** | **Betweenness centrality** | **Closeness centrality** |
| --- | --- | --- | --- |
| 1 | G (Thermovirga) - 14 | G (Smithella) - 104 | G (Thermovirga) - 0.016 |
| 2 | O (Sphingobacteriales); F (ST-12K33) - 14 | G (Thermovirga) - 97 | O (Sphingobacteriales); F (ST-12K33) - 0.016 |
| 3 | C (Anaerolineae) - 11 | G (Methermicoccus) - 91 | G (Methanospirillum) - 0.015 |
| 4 | G (Methanospirillum) - 10 | O (Sphingobacteriales); F (ST-12K33) - 88 | F (Pedosphaeraceae) - 0.015 |
| 5 | F (Synergistaceae); G (Syner-01) - 10 | G (Candidatus Methanofastidiosum) - 50 | G (Methermicoccus) - 0.014 |
| 6 | F (Pedosphaeraceae) - 10 | C (Bathyarchaeia) - 50 | F (Synergistaceae); G (Syner-01) - 0.014 |
| 7 | P (Aegiribacteria) - 10 | F (Prolixibacteraceae) - 49 | C (Anaerolineae) - 0.014 |
| 8 | G (Methermicoccus) - 9 | G (Methanospirillum) - 48 | G (Smithella) - 0.014 |
| 9 | G (Anaerolineaceae ADurb.Bin120) - 9 | G (Methanolinea) - 45 | G (Anaerolineaceae ADurb.Bin120) - 0.014 |
| 10 | F (Rikenellaceae); G (DMER64) - 9 | O (Aminicenantales) - 45 | P (Aegiribacteria) - 0.014 |
| 11 | G (Syntrophomonas) - 8 | C (Anaerolineae) - 43 | G (Syntrophomonas) - 0.014 |
| 12 | G (Smithella) - 7 | F (Methanoregulaceae) - 31 | F (Rikenellaceae); G (DMER64) - 0.014 |
| 13 | G (Candidatus Methanofastidiosum) - 6 | O (Methanosarcinales) - 30 | C (Woesearchaeia) - 0.013 |
| 14 | G (Methanobrevibacter) - 6 | F (Pedosphaeraceae) - 27 | F (Cloacimonadaceae) - 0.013 |
| 15 | C (Woesearchaeia) - 6 | P (Aegiribacteria) - 16 | G (Rikenellaceae Blvii28 wastewater sludge group) - 0.013 |
| 16 | G (Rikenellaceae Blvii28 wastewater sludge group) - 6 | F (Synergistaceae); G (Syner-01) - 11 | G (Candidatus Methanofastidiosum) - 0.013 |
| 17 | F (Cloacimonadaceae) - 6 | F (Lentimicrobiaceae) - 11 | F (Lentimicrobiaceae) - 0.013 |
| 18 | C (Bathyarchaeia) - 4 | G (Methanobrevibacter) - 8.7 | G (Methanoculleus) - 0.012 |
| 19 | G (Methanosaeta) - 4 | G (Anaerolineaceae ADurb.Bin120) - 6.6 | F (Prolixibacteraceae) - 0.012 |
| 20 | G (Methanobacterium) - 4 | G (Syntrophomonas) - 5.9 | O (Methanosarcinales) - 0.012 |
| 21 | G (Methanolinea) - 4 | F (Rikenellaceae); G (DMER64) - 5.5 | G (Methanobrevibacter) - 0.012 |
| 22 | F (Lentimicrobiaceae) - 4 | G (Methanobacterium) - 3.1 | G (Methanolinea) - 0.012 |
| 23 | G (Methanoculleus) - 3 | G (Methanosaeta) - 3 | G (Methanosaeta) - 0.011 |
| 24 | O (Methanosarcinales) - 3 | G (Methanoculleus) - 2.2 | G (Methanobacterium) - 0.011 |
| 25 | O (Aminicenantales) - 3 | C (Woesearchaeia) - 1.6 | O (Aminicenantales) - 0.011 |
| 26 | F (Methanoregulaceae) - 2 | G (Rikenellaceae Blvii28 wastewater sludge group) - 0.2 | F (Methanoregulaceae) - 0.01 |
| 27 | F (Bacteroidetes_vadinHA17) - 2 | F (Cloacimonadaceae) - 0.2 | F (Bacteroidetes_vadinHA17) - 0.01 |
| 28 | C (Anaerolineae); O (SJA-15) - 2 | O (Methanomicrobiales) - 0 | C (Bathyarchaeia) - 0.01 |
| 29 | F (Prolixibacteraceae) - 2 | F (Bacteroidetes_vadinHA17) - 0 | C (Anaerolineae); O (SJA-15) - 0.01 |
| 30 | O (Methanomicrobiales) - 1 | G (Candidatus Methanomethylicus) - 0 | O (Methanomicrobiales) - 0.009 |
| 31 | G (Candidatus Methanomethylicus) - 1 | C (Anaerolineae); O (SJA-15) - 0 | G (Candidatus Cloacimonas) - 0.008 |
| 32 | C (Methanomicrobia) - 1 | C (Methanomicrobia) - 0 | C (Methanomicrobia) - 0.008 |
| 33 | G (Candidatus Cloacimonas) - 1 | G (Candidatus Cloacimonas) - 0 | G (Candidatus Methanomethylicus) - 0.008 |

**Fig. S1** Concentrations of specific LCFA in triplicate samples from digesters R1, R2, R3, R4, R5, and R6 (quantification limit: 80 mg l^−1^). Sum of LCFA refers to the sum of palmitic, stearic and oleic acid concentrations. Note different scales on the y-axes. The LCFA measurements represent the concentrations of LCFA in the effluents which have been withdrawn from completely agitated digester.

**Fig. S2.** Biogas production was automatically recorded at 20-min intervals and evolution of biogas between feeding cycles was used for assessment of the gas production kinetics. The function, G=G_max_(1-e^-kt^), adopted from Redzwan and Banks [J Chem Technol Biotechnol 79:1174 –1178, 2004], was fitted to the daily gas production data, where G represents biogas production (ml), G_max_ maximum biogas production (ml), k coefficient rate (minute^-1^), and t time (minute). Initial biogas production rate was calculated by first derivative of the function at time zero. Half time refers to the time when G corresponds to G_max_/2. Half time gas production rate was calculated by first derivative of the exponential function at half time. 1) Start-up phase, 2) semi-continuous oleate feeding, and 3) oleate pulse feeding.

**Fig. S3** Relative abundances of 16S rRNA genes of bacterial phyla based on the average ASV reads from replicate samples for digesters R1, R2, R3, R4, R5, and R6. Numbers in brackets indicate the operational day.

**Fig. S4** Relative abundance of 16S rRNA genes associated to the genera *Methanoculleus*, *Methanobacterium*, and *Syntrophomonas* in samples collected from oleate- and sulfide-amended digester R3 (without effluent recirculation).
